# P06-05 Physical activity patterns of Hungarian women of reproductive age, preliminary study of the EUPASMOS project

**DOI:** 10.1093/eurpub/ckac095.090

**Published:** 2022-08-29

**Authors:** Viktoria Premusz, Alexandra Makai, Reka Veress, Tamas Doczi, Paulo Rocha, Pongrac Acs

**Affiliations:** 1 Faculty of Health Sciences, University of Pécs, Pécs, Hungary; 2 Hungarian Leisure Sport Association, Budapest, Hungary; 3 Department of Social Sciences, University of Physical Education, Budapest, Hungary; 4 Department of Sport and Health, Exercise and Health Laboratory, University of Lisbon, Lisbon, Portugal

**Keywords:** physical activity, women's health, EUPASMOS, RM42, GPAQ-H

## Abstract

**Background:**

Benefits of appropriate physical activity (PA) on reproductive performance of women are well known. In our study PA patterns recorded with the GPAQ-H questionnaire was corrected collected by RM42 traxial accelerometers first time in Hungary. Our aim was to examine PA patterns of women of reproductive age (RA) (15-49 years) and to evaluate their PA literacy.

**Methods:**

Data collection was conducted during February-May 2019 using quota sampling by age and gender representing the Hungarian adult population. The study comprised 345 females, 175 of them in RA (mean age 32.63±10.23 years). PA was measured for 7 consecutive days using sedentary, standing, moderate to vigorous (MVPA) and vigorous activities (VA) and daily steps scores. Data were expressed as mean ± *SD*, Spearman’s rank correlation was applied to analyse data using SPSS 24. program, where level of significance was set at p < 0.05.

**Results:**

Based on RM42 data females (N = 345) took 6483.75±3201.28 steps and spent 8.7 hours (523.00±114.31 min/day) sedentary per day and spent 6.5 hours (388.37±226.38 min/week) with MVPA and 19.12±33.98 minutes with VPA per week. The RA group (N = 175) seemed more active, 7619.32±2382.21 daily steps were measured and 229.25±304.84 min/week active transportation (walking vs cycling) self-reported. Although, they did not have sufficient knowledge on they activity patterns, significant difference (-250.92±906.95, p < 0.001) was found between self-reports (604.72±884.54 min/week) and objective measures (356.88±205.55 min/week) of MVPA and VPA was also significantly (p < 0.001) overestimated with GPAQ-H (248.78±424.50 min/week) compared to RM42 data (23.00±54.40 min/week). Negative correlation was found between age and RM42 standing (R=-0.209, p = 0.005), steps (R=-0.138, p = 0.069) and LPA (R=-0.217, p = 0.004) as well. However, the relationship was positive with MVPA (R = 0.367, p < 0.001) and VPA (R = 0.358, p < 0.001).

**Conclusions:**

Development of an integrated methodological process of PA measurement would be the main purpose of the EUPASMOS Project, but relevant information could be also obtained on certain subsamples. However, our results need to be further analysed, conclusion could be drawing, that specific interventions are needed to increase physical literacy for better understanding of appropriate intensity and level of PA, and its benefits on fertility among women of reproductive age. This work was supported by the 20765/3/2018/FEKUTSTRAT grant.

